# Serum acylcarnitines levels as a potential predictor for gestational diabetes: a systematic review and meta-analysis

**DOI:** 10.3389/fpubh.2023.1217237

**Published:** 2023-07-04

**Authors:** Hairong Luo, Zanlei Zhu

**Affiliations:** ^1^National Clinical Research Center for Metabolic Diseases, Metabolic Syndrome Research Center, Key Laboratory of Diabetes Immunology, Ministry of Education, and Department of Metabolism and Endocrinology, The Second Xiangya Hospital of Central South University, Changsha, Hunan, China; ^2^Department of Respiratory and Critical Care Medicine, Ganzhou People’s Hospital (Ganzhou Hospital Affiliated to Nanchang University/Nanfang Medical University), Ganzhou, Jiangxi Province, China

**Keywords:** GDM, plasma metabolomics, acylcarnitines, meta-analysis, predict

## Abstract

**Aims:**

Gestational diabetes mellitus (GDM) stands as a prevalent obstetric complication bearing consequential health implications for both mother and child. While existing studies have probed the alterations in acylcarnitines during GDM, an updated systematic meta-analysis is needed to consolidate these findings. Hence, this study endeavours to furnish a comprehensive systematic review and meta-analysis delineating the association between acylcarnitines and GDM, aimed at bolstering diagnostic accuracy and preventive measures against GDM.

**Methods:**

To extract pertinent studies for this meta-analysis, we meticulously scoured databases such as PubMed, Web of Science, Embase, and Cochrane Library up until May 2023. The inclusion criteria were studies contrasting plasma metabolomics between two cohorts: women diagnosed with GDM and normoglycemic pregnant women. Weighted mean differences (SMDs) and 95% confidence intervals (CIs) were calculated using random-effects models. The *I*^2^ index was employed to quantify heterogeneity amongst the studies. All meta-analyses were executed using Stata version 12.0.

**Results:**

Our meta-analysis included eight studies encompassing 878 pregnant women. The analysis disclosed that relative to normoglycemic pregnant women, women with GDM exhibited significantly elevated levels of Short-Chain Acylcarnitines (SCAC) (SMD: 0.19, 95% CI: 0.02–0.36, *I*^2^ = 71.3%). No substantial difference was discerned in fasting total carnitine levels (SMD: 0.15, 95% CI: −0.16–0.31, *I*^2^ = 68.2%), medium-chain acylcarnitines (MCAC) (SMD: 0.08, 95% CI: −0.02–0.36, *I*^2^ = 79.0%), and long-chain acylcarnitines (LCAC) (SMD: 0.04, 95% CI: −0.06–0.15, *I*^2^ = 0%). Neither funnel plot assessment nor Egger’s regression and Begg’s rank correlation tests indicated any evidence of publication bias.

**Conclusion:**

Our meta-analysis suggests that elevated levels of SCAC may heighten the risk of GDM onset. Given GDM’s deleterious impact on pregnant women and their offspring, these findings underscore the clinical imperative of managing this condition. Early surveillance of plasma metabolomic profiles, particularly serum acylcarnitine concentrations, may equip clinicians with a valuable tool for timely diagnosis and intervention in GDM.

## Introduction

Gestational Diabetes Mellitus (GDM) is a form of glucose intolerance that arises during pregnancy. This metabolic disorder is not only a significant health issue during gestation but also poses a substantial postpartum risk, with up to 50% of women with GDM history developing type 2 diabetes (T2D) within 5–10 years (1, 2). Furthermore, these individuals are more susceptible to other health issues such as non-alcoholic fatty liver disease, cardiovascular disease, and kidney disease, leading to potential premature death (3).

In recent years, metabolomics has provided valuable insights into understanding the transition from GDM to T2D (4). Among various circulating metabolites associated with diabetes, acylcarnitines (AcylCs) have garnered significant attention (5–7). These intermediate oxidative metabolites facilitate the transport of fatty acids through the mitochondrial membrane for β oxidation. Studies have noted elevated circulating levels of various acylcarnitines in confirmed T2D cases (7). However, a comprehensive understanding of changes in acylcarnitine profiles in women transitioning from GDM to T2D remains elusive.

Therefore, our study focuses on analyzing acylcarnitine levels as potential predictors for women with GDM who might progress to T2D. While several studies have addressed changes in acylcarnitines during GDM, discrepancies in findings necessitate a consolidated view to better understand the role of acylcarnitines in GDM.

Through a systematic review and meta-analysis, we aim to clarify the relationship between acylcarnitines and GDM. We believe that this study will contribute significantly to early diagnosis and intervention strategies, ultimately improving health outcomes for both mothers and infants.

## Method

### Search strategy

Two of the authors (HL and ZZ) conducted a comprehensive search of the PubMed, Embase, Web of Science, and Cochrane Library databases up to May 2023 to identify all relevant publications investigating the association between plasma acylcarnitine levels and the risk of GDM. The search terms included the following keywords: (a) gestational diabetes mellitus, (b) plasma metabolomics, (c) acylcarnitine, and (d) carnitine. In addition to electronic database searches, we also hand-searched the reference lists of included studies and relevant review articles to identify additional studies that met our inclusion criteria.

Two independent reviewers screened all titles and abstracts identified through the search strategy to determine their eligibility for inclusion in the meta-analysis. Full-text articles were retrieved for all potentially relevant studies and were reviewed independently by the same two reviewers to determine their final inclusion in the meta-analysis.

### Study selection

We included the studies in the study that met the following criteria by using the PECO/PICO (population, exposure/intervention, comparison/control, and outcome) technique.

- Population: participants who were involved in a research study that assessed the impact of plasma acylcarnitine levels on GDM.
- Exposure/Intervention: GDM present or not.
- Comparison: plasma acylcarnitine levels.
- The outcome of the study: plasma acylcarnitine levels.

Exclusion criteria.

- Studies without full text.
- *In vitro* and animal studies.
- Data of interest were not presented.
- Abstracts, commentary articles, reviews, meta-analyses, editorials, and conference presentations.

### Data extraction and quality assessment

Two of the authors (HL and ZZ) independently used a standardized data extraction form to extract the necessary information. The extracted data includes the study’s characteristics, such as the first author, publication year, study design, and country, as well as the population’s fundamental features, such as the sample size, average age, and BMI. We also extracted data on the plasma acylcarnitine levels, as well as the standard mean difference (SMD) and associated 95% confidence intervals (CIs), and *I*^2^ values. For studies that only provided the median and interquartile range (IQR), we transformed the data into the mean and standard deviation (SD) (8). Any discrepancies in the data extraction were resolved through discussion and agreement. We evaluated the quality of nonrandomized comparative studies using the Newcastle-Ottawa scale (NOS) (9).

### Statistical analysis

We used STATA software (Version 12.0; STATA Corporation, College Station, TX, United States) to perform the statistical analyses. Based on the degree of heterogeneity in the studies, either fixed-effects or random-effects models were used (fixed-effects models for *I*^2^ < 50%, and random-effects models for *I*^2^ > 50%) (10). For continuous data, we calculated the SMD with a random-effects model (DerSimonian-Laird method) and reported 95% CIs. Sensitivity analysis was conducted by excluding one study at a time through influence analysis to evaluate the stability of the results. Heterogeneity among the studies was assessed using the *I*^2^ statistic. *I*^2^ values exceeding 70% were considered indicative of extreme heterogeneity. If publication bias was present, we used Duval’s trim-and-fill method to correct it based on the properties of the funnel plot (11). Statistical significance was defined as a two-tailed value of *p* < 0.05.

## Results

### Study selection

**Figure 1:**
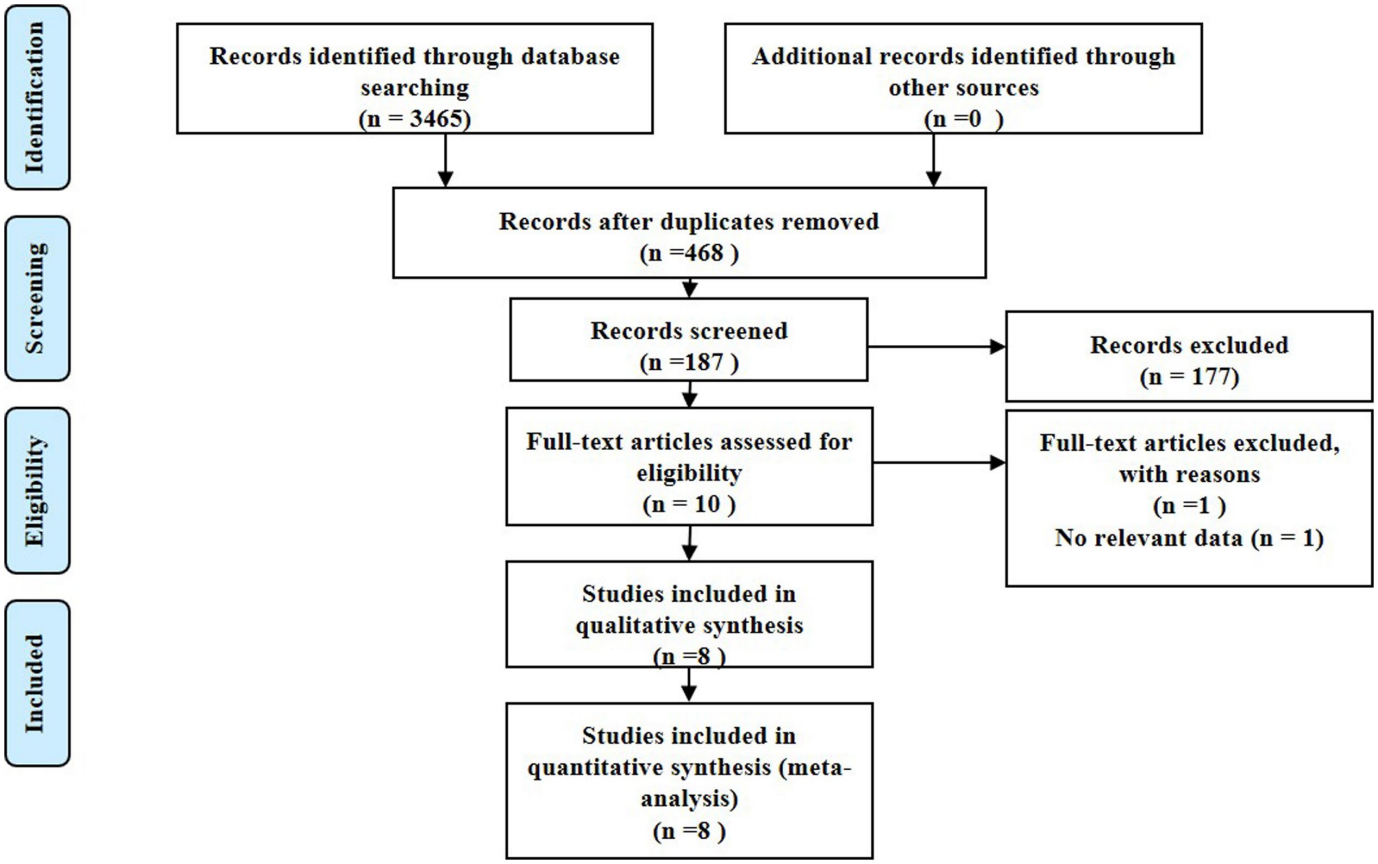
Flowchart of study selection.

This study was conducted in adherence to the Preferred Reporting Items for Systematic Reviews and Meta-Analysis (PRISMA) guidelines, eventually incorporating eight studies with a total of 878 patients that fulfilled the stipulated inclusion and exclusion criteria for the meta-analysis (12–20). Our initial literature search unearthed 3,465 articles, out of which 468 were dismissed due to duplication. A subsequent screening of titles and abstracts yielded 187 studies, with 177 more being dismissed thereafter. Following an exhaustive review of the full texts and associated articles, we further eliminated 169 studies that did not report acylcarnitine levels, thereby finalizing our selection with eight studies for our analysis. Figure 1 depicts the process of study identification and selection.

### Description of included studies

Our study embraced a total of 878 participants drawn from eight qualifying studies. This included 204 patients across three studies in Greece, 344 from two studies in Canada, 317 from two studies in China, and 13 from a single study in Brazil. The participant age range spanned from 22 to 46 years. As for the design, the study comprised two cohort studies, four case-control studies, and two prospective studies. The follow-up period for the two cohort studies were 2 years and 2–3 months, respectively. These studies were published over a span of years, from 2005 to 2022. According to the Newcastle-Ottawa Scale (NOS) scores, all studies showcased high quality. Table 1 provides a summary of the characteristics and NOS scores of the eight qualifying studies.

**Table 1:** Studies included in the meta-analysis.

Number	Author	Year	Country	Type of study	Sample size	Age (years)	BMI (kg/m^2^)	Plasma carnitine measured	NOS
1	KALLIOPI I. PAPPA	2005	Greece	Prospective study	79	Normal pregnant: 27.85 ± 4.99; GDM: 28.06 ± 5.32	Normal pregnant: 23.49 ± 5.24; GDM: 27.47 ± 5.13	McGarry and Foster’s radioisotopic assay	8
2	KALLIOPI I. PAPPA	2006	Greece	Prospective study	71	Normal pregnant: 27.85 ± 4.99; GDM: 27.84 ± 5.14	Normal pregnant:23.49 ± 5.24; GDM:27.32 ± 5.56	McGarry and Foster’s radioisotopic assay	8
3	Eleni Agakidou	2013	Greece	Case-control study	54	Normal pregnant:30.4 ± 5.6; GDM: 31.3 ± 4.9	Normal pregnant: 27.9 ± 4.8;GDM:32.3 ± 5.8	Double quadruple mass spectrometer API2000 (Applied Biosystems, Foster City, CA 94404, United States) through an HPLC system (PE200, Perkin Elmer, Southfield, MI 48075, United States)	8
4	Amina Allalou	2016	Canada	Cohort study	244	Normal pregnant: 33.1 ± 4.5; GDM:33.3 ± 5.2	Normal pregnant: 33.3 ± 8.3; GDM: 33.5 ± 8.4	p150 AbsoluteIDQ^™^ plate technology (Biocrates Life Sciences AG, Austria)	9
5	Cynthia Roy	2018	Canada	Case-control study	100	Normal pregnant: 31 ± 3.7; GDM:31 ± 3.8	Normal pregnant: 25.7 ± 5.2; GDM: 25.7 ± 5.4	Ultra-high pressure liquid-chromatography quadrupole time-of-flight mass spectrometry instrument	8
6	Huiqian Zeng	2018	China	Case-control study	196	Normal pregnant: 29.98 ± 4.50; GDM: 31.35 ± 3.77	Normal pregnant: Weight: 65.91 ± 8.61 Height:160.03 ± 4.91; GDM:Weight:67.61 ± 9.79 Height; 159.66 ± 5.00	Tandem mass spectrometry(MS/MS)	8
7	Zhongde Wang	2019	China	Case-control study	121	Normal pregnant: 29.32 ± 3.61; GDM: 30.75 ± 4.23	Normal pregnant: Weight: 58.35 ± 9.24 Height:162.13 ± 5.56; GDM:Weight:64.71 ± 10.67 Height: 162.17 ± 4.81	High performance liquid chromatography–tandem mass spectrometry	8
8	Gabriela D. A. Pinto	2022	Brazil	Cohort	13	Normal pregnant: 27 ± 5.51; GDM: 36.3 ± 10.48	Normal pregnant: 23.06 ± 3.58; GDM:30.74 ± 5.72	Acylcarnitine analysis (Cambridge Isotope Laboratories, Inc.; Massachusetts, United States)	8

### Overall analysis

#### Meta-analysis results of fasting total carnitine levels in GDM group and controls

**Figure 2:**
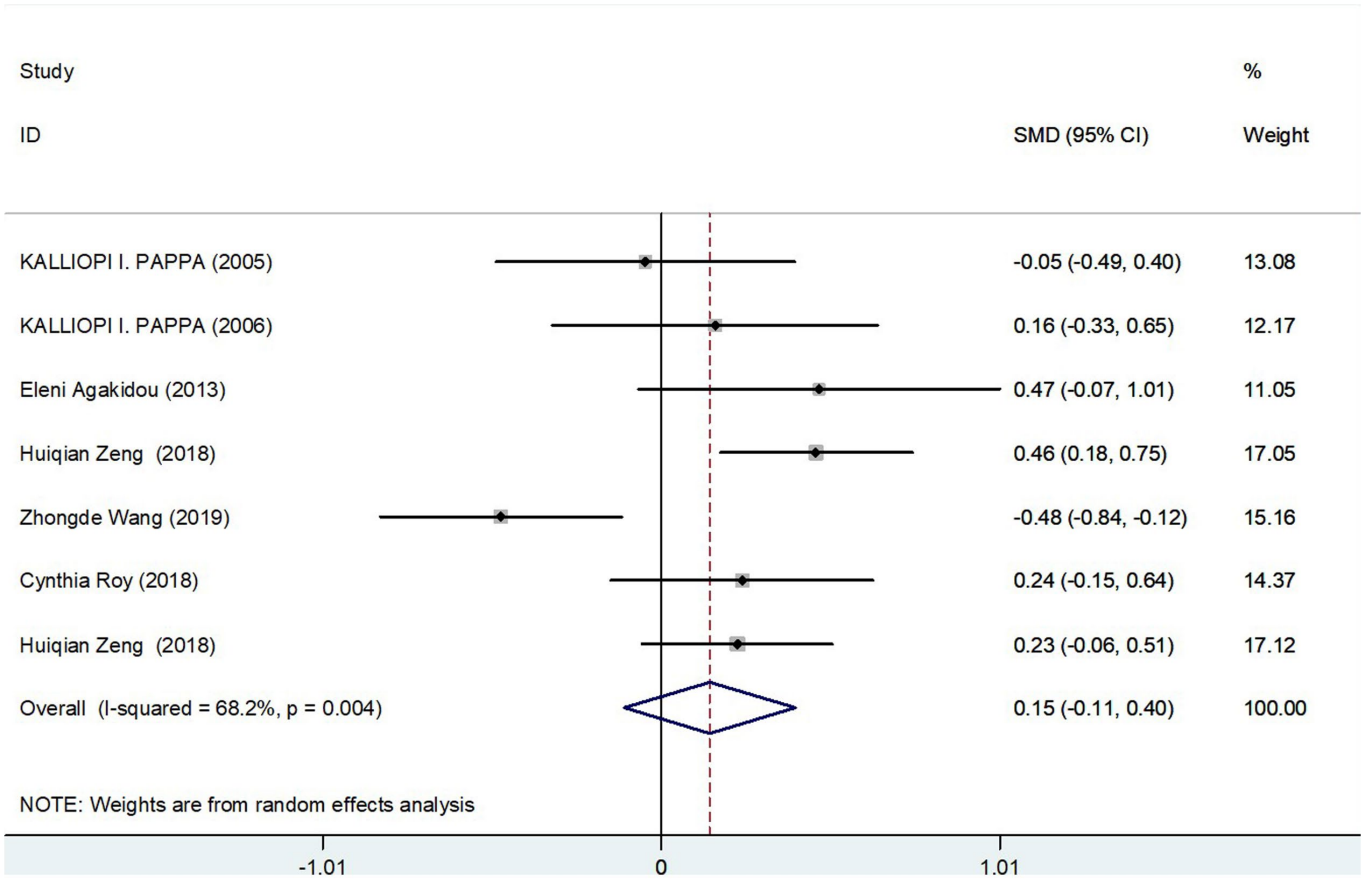
Fasting total carnitine levels in people with GDM compared to those without GDM.

**Figure 3:**
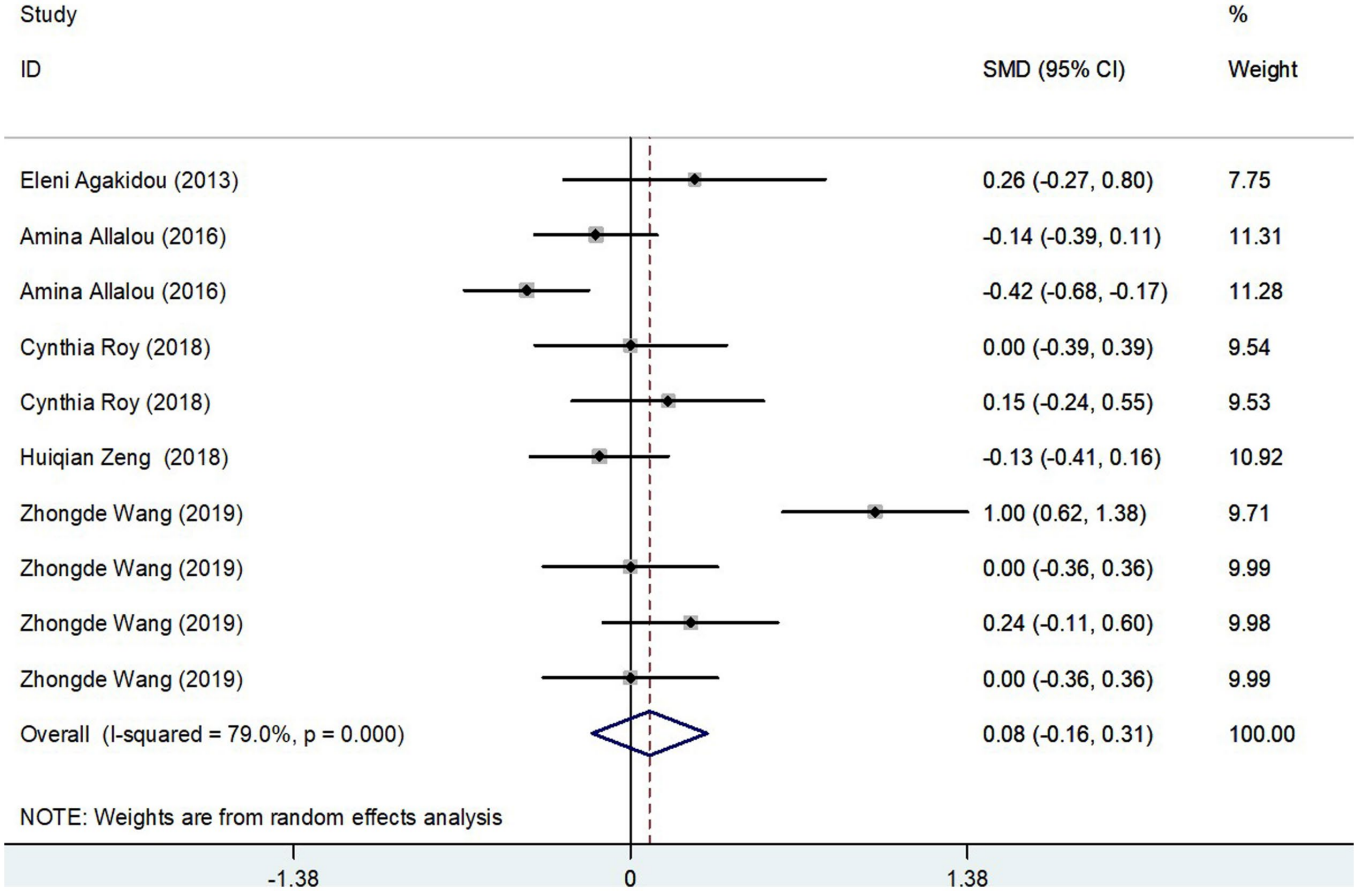
Sensitivity analyses of the SMD for **(A)** total carnitine levels, **(B)** SCAC, **(C)** MCAC, and **(D)** LCAC and GDM.

The meta-analysis incorporated seven studies with a total of 817 participants, all of which reported data on fasting total carnitine levels for both the GDM and control groups. The calculated effect size, using a random-effects model, revealed no significant differences in fasting total carnitine levels between the two groups, yielding a pooled Standard Mean Difference (SMD) of 0.15 and a Confidence Interval (CI) spanning −0.11 to 0.40 (Figure 2). Further assessments, sensitivity analysis affirmed the robustness of these results (Figure 3A). Collectively, these findings suggest an absence of significant difference in fasting total carnitine levels between pregnant women diagnosed with GDM and those without the condition.

#### Meta-analysis results of SCAC levels in GDM group and controls

**Figure 4:**
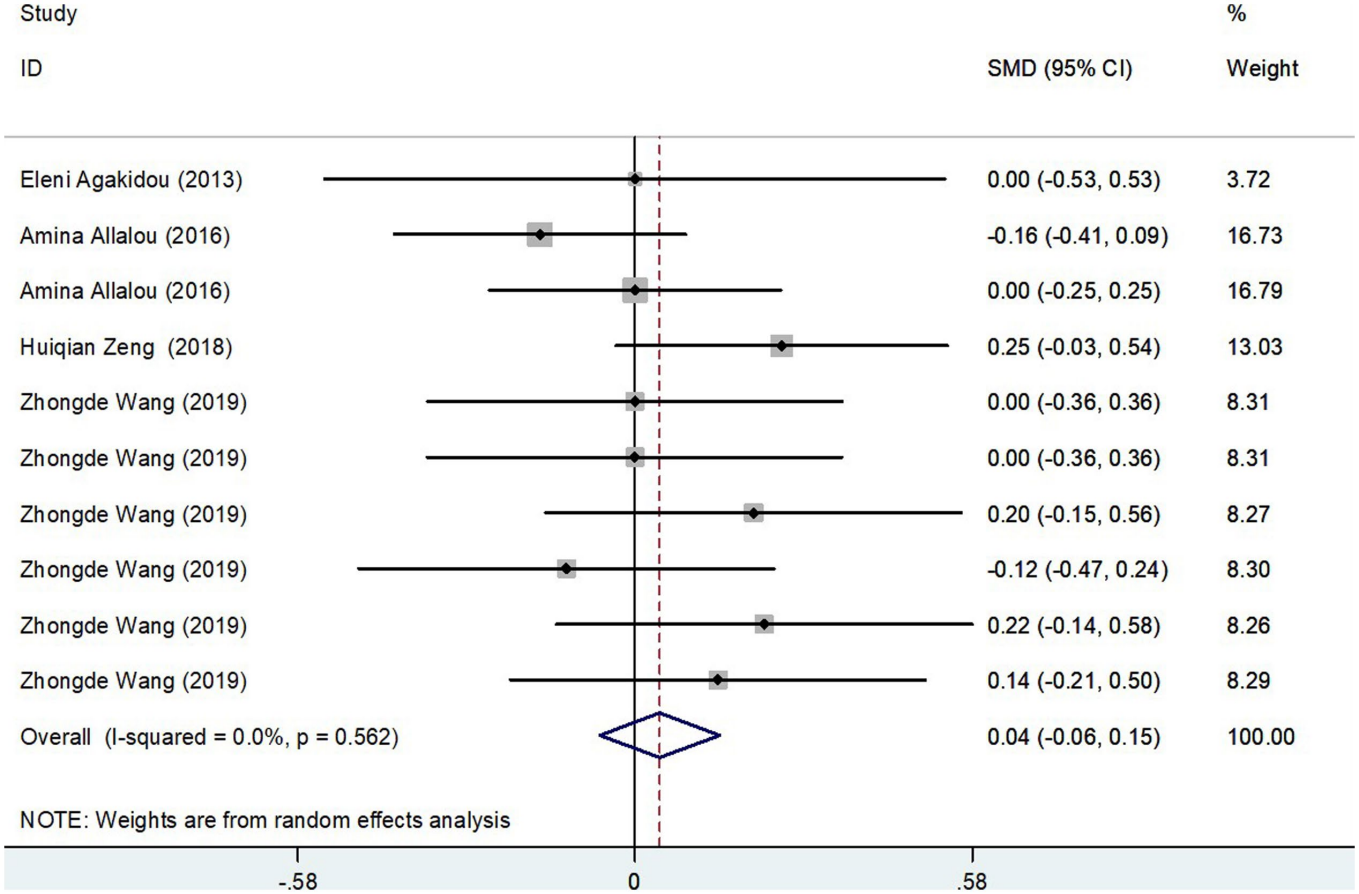
SCAC levels in people with GDM compared to those without GDM.

The meta-analysis results, derived from five studies comprising 715 participants, showed that Short-Chain AcylCarnitines (SCAC) levels were significantly higher in the GDM group compared to controls. The effect size, as determined by the random-effects model, was moderate, with a pooled Standard Mean Difference (SMD) of 0.19 and a Confidence Interval (CI) between 0.02 and 0.36 (Figure 4). However, notable heterogeneity was observed among the studies (*I*^2^ = 71.3%, *p* = 0.000). Further assessments using sensitivity analysis validated the stability of these results, with no individual study significantly skewing the overall findings (Figure 3B). Summarily, these findings suggest that pregnant patients with GDM exhibit higher SCAC levels than those without the condition.

#### Results of the meta-analysis of MCAC levels in GDM group and controls

**Figure 5:**
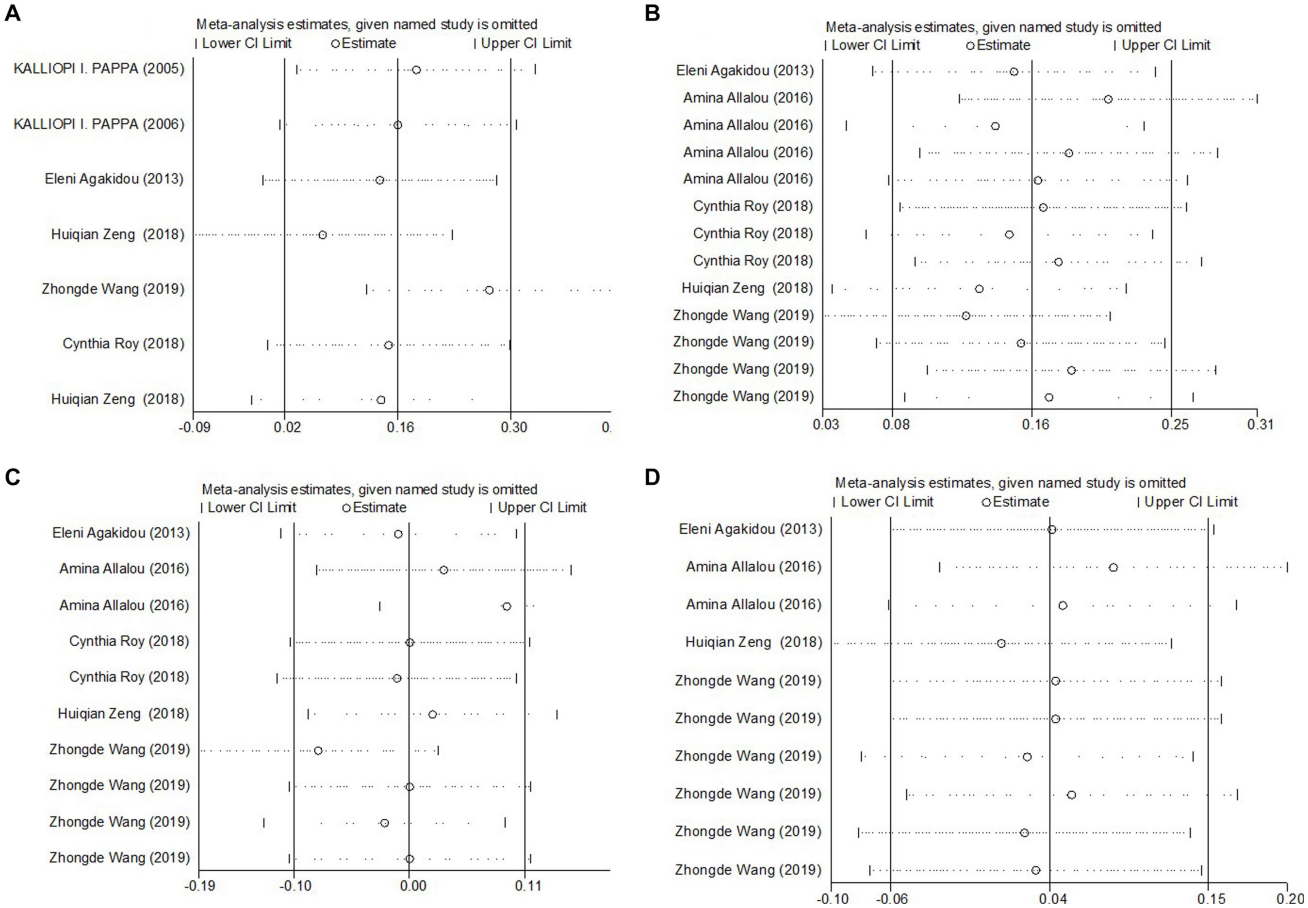
MCAC levels in people with GDM compared to those without GDM.

The meta-analysis, collating data from five studies that included a total of 715 participants, found no significant difference in Medium-Chain AcylCarnitines (MCAC) levels between the GDM group and controls. The effect size, determined using a random-effects model, demonstrated no significant variation in MCAC levels, with a pooled Standard Mean Difference (SMD) of 0.08 and a Confidence Interval (CI) between −0.16 and 0.31 (Figure 5). The heterogeneity test results indicated substantial heterogeneity (*I*^2^ = 79.0%, *p* = 0.000). Further stability of the findings was confirmed via sensitivity analysis, revealing that no single study significantly influenced the overall results (Figure 3C). Consequently, these findings imply that pregnant patients with GDM do not exhibit significantly elevated MCAC levels compared to those without GDM.

#### Results of the meta-analysis of LCAC levels in GDM group and controls

**Figure 6:**
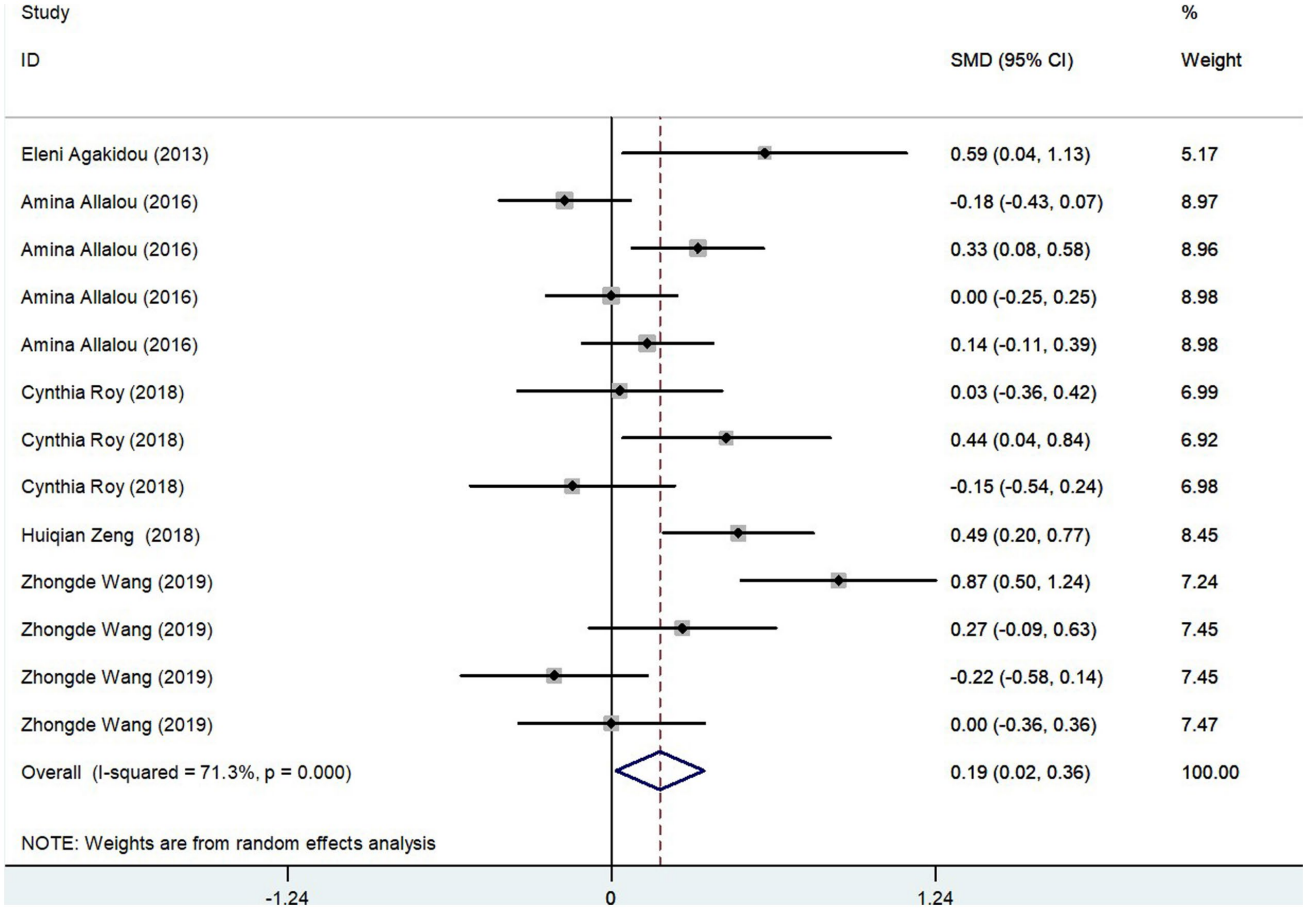
LCAC levels in people with GDM compared to those without GDM.

The meta-analysis incorporated data from four studies, including a total of 615 subjects, analyzing Long-Chain AcylCarnitines (LCAC) levels in both GDM patients and control groups. Using a random-effects model, the effect size showed no significant difference in LCAC levels, with a pooled Standard Mean Difference (SMD) of 0.04 and a Confidence Interval (CI) from −0.06 to 0.15 (Figure 6). The heterogeneity test results indicated no significant heterogeneity (*I*^2^ = 0.0%, *p* = 0.562). Further confirmation of the robustness of the findings was provided by sensitivity analysis, illustrating that no single study had a significant impact on the overall results (Figure 3D). Thus, these results suggest that there is no significant difference in LCAC levels between pregnant women with GDM and those without GDM.

#### Results of the meta-analysis of others parameters in GDM group and controls

The meta-analysis included three studies, with a total of 204 participants, examining fasting free-carnitine levels between the GDM and control groups. The derived effect size from the random-effects model indicated no significant changes in fasting free-carnitine levels, with a pooled Standard Mean Difference (SMD) of 0.26 and a Confidence Interval (CI) of −0.97 to 1.47 (Table 2). The heterogeneity test revealed considerable heterogeneity (*I*^2^ = 94.10%, *p* = 0.000). Similarly, three studies encompassing 204 subjects were included in the meta-analysis assessing fasting acylcarnitine levels in the GDM and control groups. The effect size from the random-effects model again showed no significant changes in fasting acylcarnitine levels (pooled SMD: 0.22, CI: −0.50 to 0.95) (Table 2), with notable heterogeneity (*I*^2^ = 84.20%, *p* = 0.001). Two studies, consisting of 209 subjects, provided data on the ratio of total acylcarnitine to free carnitine in the GDM group and controls. The effect size derived from the random-effects model displayed no significant changes in this ratio (pooled SMD: −0.21, CI: −0.49 to 0.06) (Table 2). Here, the heterogeneity test results indicated no significant heterogeneity (*I*^2^ = 0.0%, *p* = 0.490). Collectively, these results suggest that there may not be a significant difference in fasting free-carnitine, fasting acylcarnitine levels, and the ratio of total acylcarnitine to free carnitine between pregnant women with GDM and those without GDM.

**Table 2:** Meta-analysis of other parameters (fasting free carnitine, fasting acylcarnitine, and total acylcarnitine/free carnitine).

Others parameters	Study	Number of patients	SMD	95% CI	*I* ^2^	*p*
Fasting free-carnitine	3	204	0.26	(−0.97, 1.47)	94.10%	0
Fasting acyl-carnitine	3	204	0.22	(−0.50, 0.95)	84.20%	0.001
Total acylcarnitine/Free carnitine	2	209	−0.21	(−0.49, 0.06)	0.00%	0.49

## Discussion

### Association between GDM and plasma acylcarnitines levels

In our study, in GDM, most of the changed acylcarnitines is medium-chain acylcarnitines, while long-chain acylcarnitines have not changed much. Therefore, it can be speculated that in early diabetes, cells are damaged to a minimum, so long-chain acylcarnitines can be reduced to short and medium-chain acylcarnitines, leading to the accumulation of these acylcarnitines first. As the damage progresses, long-chain acylcarnitine can no longer be metabolized, leading to an increase in long-chain acylcarnitine in patients with dominant diabetes (16). Therefore, based on the analysis results, we can hypothesize that the short- and medium-chain acylcarnitine may be an early sign and driving factor of β-cell dysfunction during the transition from gestational diabetes to T2D (14). Validation can be used as an early diagnosis method to better predict GDM (13). At the same time, it can be used as a treatment method to slow down the condition by controlling the content of short and medium chain acylcarnitine and as a long-term detection index.

In the following text, we will discuss the potential mechanisms by which acylcarnitines may contribute to the development of GDM. First of all, elevated levels of plasma acylcarnitines have been implicated in the development of GDM, a common complication of pregnancy. Acylcarnitines are derived from the breakdown of fatty acids and their accumulation in the blood is indicative of impaired fatty acid oxidation (21). This impairment can lead to an excess of glucose in the bloodstream, which can contribute to insulin resistance and ultimately (22), the development of GDM. Secondly, studies have shown that elevated levels of specific acylcarnitines, such as C3 and C5, are associated with an increased risk of GDM. These acylcarnitines are thought to interfere with insulin signalling and impair glucose uptake by cells (23), leading to elevated blood glucose levels. Additionally, they may promote inflammation and oxidative stress (24), which further contribute to insulin resistance. Finally, high levels of acylcarnitines have been shown to impair pancreatic beta cell function (25), which is critical for maintaining glucose homeostasis. Beta cells produce insulin, which regulates blood glucose levels, and impairment of these cells can lead to GDM. In summary, elevated levels of plasma acylcarnitines may contribute to the development of GDM through their effects on insulin signaling, glucose uptake, inflammation, oxidative stress, and beta cell function. Further research is needed to fully understand the mechanisms underlying this relationship and to identify potential therapeutic targets for preventing and treating GDM.

In the results of our meta-analysis, considerable heterogeneity is observed particularly in the assessment of SCAC and MCAC levels in women with GDM versus normoglycemic pregnant women. The reported *I*^2^ values, which signify the percentage of the total variation across studies due to heterogeneity, are high for these two groups. There are several potential reasons for this high degree of heterogeneity: 1. Differences in Study Populations: the included studies may have involved diverse populations with different demographic and clinical characteristics such as age, ethnicity, body mass index, and gestational age. These factors can potentially influence acylcarnitine levels and GDM incidence. 2. Variations in GDM Diagnosis: different criteria for diagnosing GDM across various countries and institutions might contribute to the inconsistency in the included studies. Some may follow the International Association of Diabetes and Pregnancy Study Group (IADPSG) criteria, while others may use the World Health Organization (WHO) or American Diabetes Association (ADA) guidelines. 3. Differences in Sample Collection and Handling: acylcarnitine measurements might be influenced by pre-analytical factors such as sample collection, processing, and storage conditions. Also, the use of different assay techniques across studies may yield different results. 4. Variation in Dietary Intake: the dietary pattern, which can influence metabolism and thus the levels of acylcarnitines, might not be similar in different studies. It is a potential confounder that could contribute to heterogeneity. 5. Genetic Factors: genetic variants that affect acylcarnitine metabolism could also contribute to heterogeneity, especially given the ethnic diversity that is likely present across the studies included in the meta-analysis. These perspectives highlight the complexity of interpreting and synthesizing data from various studies into a meta-analysis.

### Strengths and limitations

The strength of this article is its pioneering meta-analysis that sheds light on the relationship between acylcarnitines and GDM. The diagnosis of GDM typically involves screening for abnormal glucose levels during pregnancy. However, recent studies have shown that testing serum acylcarnitine levels may offer additional benefits in predicting the development of GDM. Acylcarnitines are metabolites that play a crucial role in the transport of fatty acids into the mitochondria for energy production. Elevated levels of certain acylcarnitines have been found to be associated with insulin resistance and glucose intolerance, both of which are key features of GDM. By testing serum acylcarnitine levels early in pregnancy, clinicians may be able to identify women who are at higher risk for developing GDM and intervene with lifestyle modifications or medical therapy before the onset of the condition. This could lead to better outcomes for both the mother and baby, including reduced risk of macrosomia, neonatal hypoglycemia, and cesarean delivery. Overall, while screening for abnormal glucose levels remains the primary method for diagnosing GDM, testing serum acylcarnitine levels may provide valuable additional information that could ultimately improve outcomes for women with this condition.

Despite the findings, this study has some limitations that warrant attention. Firstly, the quantity of studies included in the analysis is limited, potentially undermining the conclusiveness of our findings regarding the influence of short- and medium-chain acylcarnitines on GDM. Secondly, our analysis did not consider acylcarnitine levels throughout all stages of pregnancy. This omission might restrict our capacity to pinpoint the earliest possible period during which these acylcarnitines could be effectively detected. Future investigations could thus aim to devise more comprehensive studies that track changes in acylcarnitine metrics at varying pregnancy stages. Thirdly, our meta-analysis is inherently reliant on the availability of published data, and potentially relevant unpublished studies may not have been included in our analysis, possibly introducing bias into our results. Additionally, a meta-analysis, by design, cannot confirm causality, and it is plausible that other, unaccounted-for factors might also contribute to the onset of GDM. Early blood tests may predict the onset of disease, offering prospective information, while case-control studies collect data after the onset of disease, providing retrospective information. These two types of studies have fundamentally different methodologies and aims, which should be taken into account during data interpretation. Lastly, the precise acylcarnitine levels that could contribute to GDM remain undefined. While our study provides valuable insights into the association between acylcarnitines and GDM, further exploration is necessary to fully comprehend this relationship. In conclusion, while our meta-analysis offers meaningful insights into the potential predictive utility of serum acylcarnitine levels for GDM, the limitations of this approach need to be considered, and the results interpreted cautiously. Additional studies are warranted to further probe this association and to establish the clinical relevance of serum acylcarnitine levels as a predictor of GDM.

## Conclusion

In conclusion, our analysis reveals that individuals with GDM show elevated serum concentrations of medium-chain acylcarnitines. This observation underscores the potential diagnostic and predictive value of acylcarnitine analysis in the context of GDM. Nevertheless, owing to the relatively scarce number of clinical studies available, the current dataset might be inadequate to thoroughly elucidate the impact of medium-chain acylcarnitines on GDM. Future investigations should place emphasis on more precise temporal measurements during pregnancy and aim to identify the most pertinent markers among the plethora of acylcarnitine measures at our disposal.

## Data availability statement

The original contributions presented in the study are included in the article/Supplementary material, further inquiries can be directed to the corresponding author.

## Author contributions

ZZ coordinated the study. HL conceived of the study, along with ZZ contributed to the study design, literature search, figures, statistical analysis, data synthesis of outcomes, and drafted and edited the final paper. All authors contributed to the article and approved the submitted version.

## Conflict of interest

The authors declare that the research was conducted in the absence of any commercial or financial relationships that could be construed as a potential conflict of interest.

## Publisher’s note

All claims expressed in this article are solely those of the authors and do not necessarily represent those of their affiliated organizations, or those of the publisher, the editors and the reviewers. Any product that may be evaluated in this article, or claim that may be made by its manufacturer, is not guaranteed or endorsed by the publisher.
